# Targeted remodeling of breast cancer and immune cell homing niches by exosomal integrins

**DOI:** 10.1186/s13000-020-00959-3

**Published:** 2020-04-18

**Authors:** Phyoe Kyawe Myint, Eun Jeong Park, Arong Gaowa, Eiji Kawamoto, Motomu Shimaoka

**Affiliations:** 1 Department of Molecular Pathobiology and Cell Adhesion Biology, Mie University Graduate School of Medicine, 2-174 Edobashi, Tsu, Mie 514-8507 Japan; 2 Emergency and Critical Care Center, Mie University Hospital, Tsu, Mie 514-8507 Japan

**Keywords:** Integrin, Exosome, Cell adhesion, Chemokine, Inflammation, Metastasis, Microenvironment

## Abstract

Exosomes represent an important subset of extracellular vesicles involved in inter-cellular communications in health and diseases. Exosomes secreted from cancer and immune cells travel to the specific tissues containing homing niches. The exosomes reaching the niches dynamically modify the gene expression and molecular architectures of the homing niche micro-environments. Cell adhesion molecule integrins regulate the tissue-specific homing patterns of not only cancer and immune cells, but also of the exosomes secreted from those cells. The exosome-mediated remodeling of the homing niches would affect immune lymphocyte migration and host defense, as well as cancer metastasis, thereby representing a potential therapeutic target.

## Main text

### Immune lymphocyte trafficking

Lymphocytes represent a key player in the adaptive immunity that protects the host from pathogens and they act in an antigen-dependent manner. To function effectively, lymphocytes do not migrate randomly in the body, but rather are regulated in order to traffic to specific tissues. Such a mechanism, termed tissue-specific homing, has been most extensively documented in gut tissues [1]. Naïve T-lymphocytes that encounter antigens in the gut lymph nodes are imprinted to home back to the gut tissues as gut-tropic effector/memory T-lymphocytes, which are then reactive to the antigen sampled in the gut lymph nodes [1]. This gut tissue-specific homing serves as a mechanism to maximize the likelihood that those T-lymphocyte clones reactive to pathogens growing in the gut could then physically counter them there. By this means they could mount an effective adaptive immunity in intestinal mucosal tissues [1].

Gut tissue-specific homing is made possible by the presence of homing niches, a microenvironment that supports lymphocyte migration to, and retention in, gut tissues [2]. High endothelial venule (HEV) endothelial cells, which are specialized endothelial cells in gut tissue post-capillary venules, are centered in the homing niche. Here, they generate a chemokine- and adhesion molecule-milieu to attract gut-tropic lymphocytes. Specifically, HEV endothelial cells express chemokine C-C motif chemokine ligand 25 (CCL25) and adhesion molecule mucosal addressin cell adhesion molecule-1 (MAdCAM-1), which support the migration of gut-tropic lymphocytes expressing the chemokine receptor C-C motif chemokine receptor 25 (CCR9). The latter is signaled by CCL25 and integrin α4β7, which bind to MAdCAM-1 [2].

**Fig. 1:**
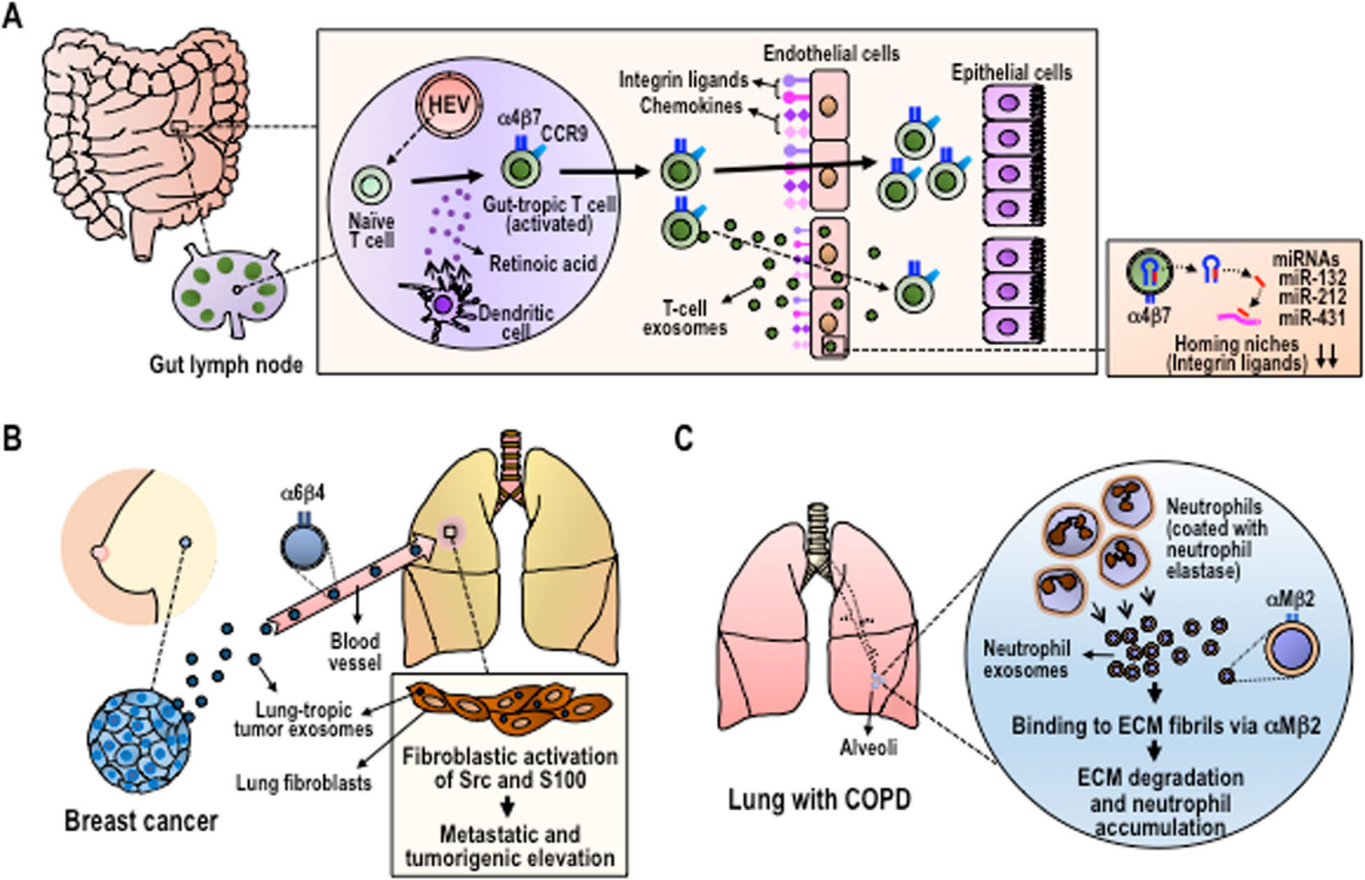
Integrin-targeted exosome trafficking to vascular endothelial cells and tissue ECM, which leads to the remodeling of cancer and immune cell homing niches. (**a**) Integrin α4β7-expresing T cell exosomes target HEVs in the gut, where they diminish homing niches by microRNAs-132, 212, and 431. (**b**) Integrin α6β4-expresing breast cancer exosomes target the laminin ECM in the lung, where they cultivate the homing niches by eliciting inflammation through Src and S-100 activation. (**c**) Integrin αMβ2-expresing neutrophil exosomes target multiple ECMs in the lung, thereby damaging alveoli structures and promoting further neutrophil accumulation by sustained neutrophil elastase proteolytic activities

MAdCAM-1 expression is developmentally regulated and, in healthy adults, remains stable and at limited levels in the gut [3]. Transcriptional factor Nirenberg-Kim (NK) 2 homeobox 3 (NKX2.3) is critical for the developmental regulation of MAdCAM-1 expression in the gut, as NKX2.3 null mice lack MAdCAM-1 expression. The signaling cascade elicited by inflammatory mediators upregulates MAdCAM-1 expression; for example, in the gut tissues of inflammatory bowel diseases, MAdCAM-1 expression is augmented, thereby promoting the accumulation of aberrantly activated T-lymphocytes expressing integrin α4β7. The recent work by Park et al. [4] revealed a new regulatory mechanism driven by exosomes secreted from the gut-tropic T lymphocytes that suppresses MAdCAM-1 expression in HEVs (Fig. 1a). This results in a remodeling of the homing niche in order to fine-tune the tissue-specific influx of activated lymphocytes.

### Exosomoal regulation of lymphocyte homing

Exosomes are lipid bilayer biological nanoparticles that are generated by the cellular endosomal pathway and which are secreted to extracellular spaces. As exosomes encapsulate microRNAs, enzymes and mediators, they act as an important means of intercellular communication between not only neighboring cells via local diffusion, but also between distant cells via systemic dissemination through the blood circulation [5, 6] **(**Fig. 1). T lymphocytes activated by encounters with antigen-bearing dendritic cells in the gut lymph nodes upregulate the expression of integrin α4β7, thereby promoting the ability to bind to MAdCAM-1 on HEVs, which is a critical step towards acquiring gut tropism [1]. Simultaneous to acquiring the ability to better home to the gut, gut tropic lymphocytes secrete exosomes that carry high levels of integrin α4β7 on the surface. Exosomal α4β7 integrins are functionally active and remain competent for binding to MAdCAM-1 [4]. When injected into mice, α4β7 integrin-expressing T-cell exosomes are preferentially distributed to the gut mucosa in an integrin-dependent manner, as is observed with gut-tropic T lymphocytes [4]. Remarkably, α4β7 integrin-expressing T-cell exosomes are enriched with a set of microRNAs that target NKX2.3, thereby suppressing MAdCAM-1 expression [4]. Treatment of mice with the α4β7 integrin-expressing T-cell exosomes inhibited subsequent T-lymphocyte homing to the gut, especially to the small intestine, by suppressing MAdCAM-1 expression and other homing niche components such as the chemokines CCL25 and C-C motif chemokine ligand 28 (CCL28) [4]. These results support the concept that α4β7 integrin-expressing T-cell exosomes are capable of preferentially targeting gut HEV endothelial cells functionality, thereby diminishing the capacity of the homing niches to support migration and retention of gut-tropic lymphocytes (Fig. 1a**)**. The T-cell exosome-mediated down-regulation of the homing niche might act as an important mechanism to counter-balance the gut-specific migration of gut-tropic T lymphocytes in order to maintain tissue homeostasis in the homing niches.

Like other cellular integrins, the ability of those α4β7 integrins expressed on lymphocytes to bind ligand MAdCAM-1 is dynamically regulated by intracellular signaling cascades that culminate in the binding to the integrin cytoplasmic domain by an adaptor protein talin [7]. This dynamic and reversible binding of talin enables the versatile affinity regulation of integrins. The absence of the intracellular signaling cascade due to the loss of a talin co-activator kindlin has been shown to generate non-adhesive leukocyte integrins, which has been observed in a primary immune deficiency known as leukocyte adhesion deficiency type III. Soe et al. has shown that talin-2, one of two talin isoforms, is critical for the ability of the exosomal integrins α4β7 and αLβ2 to bind ligands [7]. Notably, these results have demonstrated that even within exosomes measuring ~ 150 nm diameter, parts of the integrin-signaling cascades operate to support exosomal integrin activities [7].

### Exosomal regulation of cancer metastasis

Cancer cell-secreted exosomes have been shown to constitute one of the tumor-derived factors that contribute to the formation of pro-tumor micro-environments such as increased vascular permeability, angiogenesis and chronic inflammation [8]. In contrast to those T-cell exosomes that play a negative remodeling role and diminish the existing homing niches, cancer exosomes have been shown to positively impact remodeling, thereby inducing homing niches to aid subsequent cancer metastasis (Fig. 1b). Hoshino et al. [8] has shown that breast cancer exosomes expressing high levels of integrin αVβ5 are distributed to liver tissue containing a fibronectin-enriched extracellular matrix (ECM), whereas those expressing high levels of integrin α6β4 are distributed to the lung tissue containing an ECM enriched with laminin [8]. In both types of breast cancer exosomes, exosomal integrins not only target the ECM proteins in the remote tissues, but are also are themselves delivered to the targeted cells (i.e., liver and lung stromal cells), in which they activate Src-kinase signaling, thus leading to the induction of a proinflammatory S-100 gene [8] (Fig. 1b). Indeed, chronic inflammation is known to contribute to cancer metastasis. Thus, the integrin-guided preferential distribution of cancer exosomes determines which specific organs might suffer the cancer exosome-mediated initiation of inflammation. This, in turn, would “cultivate” a microenvironment permissive to subsequent cancer homing, retention, and growth, alternatively known as a pre-metastatic niche (Fig. 1b).

Exosomal integrins are also involved in the remodeling of the tumor microenvironments of primary sites. For example, prostate cancer cells secrete αVβ3 and αVβ6 integrin-enriched exosomes that serve as the cargo for horizontally transferring those integrin proteins targeting neighboring non-tumorigenic cells. These exosomally transferred αVβ3 and αVβ6 integrins are functionally active and appear on the surface of neighboring non-tumorigenic cells, thereby converting otherwise inert cells to migratory and potentially inflammatory phenotypes [9]. These horizontal transfers of αV integrin proteins could occur in those stromal cells surrounding cancer cells, such as tumor-associated fibroblasts and tumor-associated endothelial cells, thereby remodeling the local micro-environment towards pro-tumor conditions that would allow cancer cells to grow invasively.

### Exosomal regulation of COPD pathology

Targeting ECM proteins by exosomal integrins facilitates the remodeling of microenvironments not only in the remote tissues, such as is observed in breast cancer exosomes, but also in local tissues – e.g., those neutrophil exosomes released into the inflamed lung [10] (Fig. 1c). In chronic obstructive pulmonary disease (COPD) and bronchopulmonary dysplasia, those activated neutrophils that have accumulated in the lung release integrin αMβ2-expressing exosomes, which are surface coated with neutrophil elastase [10]. As integrin αMβ2 binds to promiscuous ECM ligands and as neutrophil elastase express broad substrate specificities, neutrophil exosomes demonstrate the ability to robustly proteolyze ECM proteins [10]. In addition, co-existence with integrin αMβ2 on the surface of exosomes helps to protect exosomally associated neutrophil elastase against the neutralizing activities of α1-anti-trypsin present in the lung [10]. In this way, neutrophil exosomes continue to carry out activities to destroy the lung alveoli, thereby giving rise to the pathological tissue remodeling that causes further migration of activated neutrophils to the lung (Fig. 1c). These dynamics reinforce the vicious cycles of inflammatory tissue damage that leads to pulmonary emphysema.

Increasing evidence, like that provided here, points to the pivotal role played by exosomal integrins in the targeted remodeling of micro-environments, which strongly impacts subsequent cancer and immune cell trafficking to specific tissues. Further investigations are needed to aid the development of novel therapeutic approaches geared towards interfering with exosomal integrins. By this means, inflammation in cell micro-environments can be alleviated and physiologic tissue homeostasis restored [5, 6].

## Data Availability

NA

## Abbreviations

CCL25: C-C motif chemokine ligand 25
CCL28: C-C motif chemokine ligand 28
CCR9: C-C motif chemokine receptor 9
COPD: Chronic obstructive pulmonary disease
ECM: Extracellular matrix
HEV: High endothelial venule
MAdCAM-1: Mucosal Addressin Cell Adhesion Moecule-1
NKX2.3: Nirenberg-Kim (NK) 2 homeobox 3
